# Methodologies and methods for research on non‐pharmacological interventions for dementia: Gaps and state‐of‐the‐art evidence

**DOI:** 10.1002/alz70858_100891

**Published:** 2025-12-25

**Authors:** Federica D'Andrea, Sara Laureen Bartels, Maud Graff, The INTERDEM Methodology Taskforce

**Affiliations:** ^1^ The Geller Institute of Ageing and Memory, University of West London, London, London, United Kingdom; ^2^ Maastricht University, Maastricht, Limburg, Netherlands; ^3^ Department of Rehabilitation, Radboudumc, Radboud University Medical Centre Nijmegen, Nijmegen, Gelderland, Netherlands; ^4^ Department of IQ Health, Radboudumc Alzheimer Center, Radboud University Medical Center Nijmegen, Nijmegen, Gelderland, Netherlands

## Abstract

**Background:**

The Medical Research Council (MRC) framework is a widely cited source and guides the development, evaluation, and implementation of complex interventions, such as non‐pharmacological interventions for dementia. It outlines core components (e.g., consider context, involve stakeholder involvement, utilize program theory) across phases (i.e., development, feasibility/piloting, evaluation, implementation). The updated version (Skivington et al., 2021) emphasizes the investigation of underlying mechanisms of change, societal impact, and intervention sustainability. However, there appears to persist a predominant reliance on Randomized Controlled Trials (RCTs) for evidence, often overlooking contextual factors and the dynamic complexities in dementia care settings, thus limiting insights into the mechanisms underlying changes. While new methodological approaches in dementia research are emerging, it is unclear how they match the MRC framework and address its core elements.

**Aims:**

The METHODEM project aims at i) discussing methodological gaps in research on non‐pharmacological interventions for dementia, and ii) providing a state‐of‐the‐art overview of methodologies and methods used across the MRC framework phases.

**Methods:**

First, the INTERDEM Methodology Taskforce consisting of multi‐disciplinary, international researchers shared an opinion piece, discussing methodological gaps in the field. Second, a scoping review is ongoing, mapping methodologies and methods used for the development, evaluation, and implementation of non‐pharmacological intervention in dementia care since 2000 onto the MRC framework.

**Results:**

Viewed through the current MRC framework lens, a collection of relevant and novel methodologies and methods (e.g., Realist evaluation, Co‐production research approaches, Pragmatic Trials, Theory of Change) within the growing field of non‐pharmacological interventions for dementia were identified and discussed. Critical reflections were drawn from various dementia research projects. First insights from the review are expected by the summer 2025.

**Conclusions:**

The identified relevant and novel methodologies and methods for research on non‐pharmacological interventions in dementia care showcase research approaches that adhere to multiple core components of the MRC framework. Findings will inform a Delphi study (ARCOM‐24‐1250087 awarded) to reach a consensus on the appropriate approaches to develop, implement, and evaluate interventions. The goal is to stimulate a methodological debate and to reduce the gap between research and practice, and ultimately advance best practice in the field.

